# Supplementary material for: Are carbon emissions trading and green financial instruments synergistic? -Comprehensive quantitative research based on content analysis
Source: PLoS One. 2024 Mar 7;19(3):e0298601. doi: 10.1371/journal.pone.0298601 (PMC10919601; doi:10.1371/journal.pone.0298601)
Supplement: S1 Data — (ZIP) [file pone.0298601.s001.zip › Data/地级市Z_F工作报告.zip!/░▓╗╒/╦▐╓▌╩╨/└┤╫╘╣1⁄2╓┌║┼í╛┬φ┐╦╩2╛▌═°í┐_2008.docx]

2008年1月4日宿州市第二届人民代表大会第五次会议

市长  吴旭军

各位代表：

现在，我代表市人民政府，向大会报告工作，请予审议，并请市政协委员和其他列席人员提出意见。

一、2007年工作回顾

刚刚过去的一年，是我市立足新起点、推动经济社会又好又快发展的一年。在市委的正确领导下，市政府团结带领全市人民，全面落实科学发展观，按照市第三次党代会的战略部署，紧紧围绕“全面追赶、重点突破、局部超越”的总体要求，抢抓机遇，锐意进取，圆满实现了市二届人大四次会议确定的主要目标。预计：

——全市生产总值突破400亿元，达到425亿元，增长11%以上。

——财政总收入突破20亿元，达到21亿元，增长25%。

——固定资产投资突破百亿元，达到116亿元，增长30%。

——全社会消费品零售总额突破百亿元，达到110亿元，增长17%。

——城镇居民人均可支配收入突破万元，达到10500元，农民人均纯收入突破3000元。

一年来，市政府牢固树立以经济建设为中心，始终坚持加快发展、和谐发展、引领发展，各项工作都取得了显著成效。

（一）工业总量快速扩张，支撑作用初步呈现。强力推进工业三年倍增，工业经济发展速度、效益大幅提升，二产对经济增长的贡献率提高6.3个百分点。规模以上工业增加值完成75亿元，增长30%，净增规模以上工业企业93户。工业综合效益指数达140%，提高12个百分点。完成工业技改投资40亿元，增长22.7%，一批重点技改项目建成投产。加快名牌产品培育，“皖王”小麦粉、雪龙棉浆等5个产品分别获中国和安徽名牌产品称号。民营经济发展迅猛，私营企业、个体工商户新增900家和6000个。积极破解中小企业融资难题，为680户中小企业建立了信用档案，284户中小企业成为金融机构重点扶持对象，新增贷款近8亿元，增量与增幅均创近年新高。

（二）新农村建设扎实推进，县域经济竞相发展。克服多种自然灾害影响，农村经济持续增长。全年粮食总产372.7万吨，再创历史新高。农业结构进一步优化，农产品优质率稳步提高，畜牧规模养殖比重和效益大幅攀升。农业产业化经营迅速发展，市级以上龙头企业达113家，营销收入85亿元，增长43%，营销收入超亿元企业达25家；产业化基地新增129万亩，农民合作经济组织发展到440个。农产品质量安全水平逐步提高，2个国家级农业标准化示范区通过国家验收，5个农业标准化生产基地分别通过国家、省评审认定。培训农民4.3万人，劳务输出总量突破130万人，劳务结构明显改善。新农村建设加快推进，“村村通”道路工程建成1160公里，农村用电全部实现同网同价；“十镇百村”示范工程成效初显，饮水安全、户用沼气等十件实事顺利推进。万里绿色长廊二期建设、农机化综合水平保持全省领先。全面落实支农惠农政策，各类补贴资金按时足额发放。农村综合改革全面启动，农业普查扎实进行。对县域经济发展的支持力度加大，各县区规模以上工业增加值、固定资产投资、财政收入等主要经济指标增幅均高于全市平均水平，为全市经济发展提供了有力支撑。

（三）招商规模大幅跃升，开放领域不断拓宽。深入实施东向发展战略，强化专业招商和重点领域招商，到位内资突破100亿元，增长70%，增幅位居全省前列；实际利用外资5300万美元，增长63%，落地千万元以上项目437个，其中亿元以上项目57个。组团参加中部博览会和国际徽商大会，成功举办第四届中国宿州黄淮海地区农博会、中国果蔬加工砀山论坛及苏浙四市项目推介会。招商平台建设加快，宿州经济开发区路网日趋完善，生产总值实现翻番；各县区开发区共落实开行贷款4.6亿元，基础设施建设累计投入2.2亿元，落地投资千万元以上项目62个，承载能力明显增强；黄圩、朱仙庄、黄楼、尤集、张庄寨等一批特色鲜明的乡镇工业功能区初具雏形，集聚效应开始显现。对外贸易平稳增长，外贸进出口4800万美元，增长16%。

（四）重点项目建设卓有成效，发展后劲显著增强。在国家宏观调控的新形势下，牢牢抓住项目建设不放松，扎实推进“6＋3”工程，一批事关全局的重大项目取得历史性突破。华电一期2×600MW机组并网发电，新锦丰集团、皖王面粉、瀚森荷金来、海升果业、虹光玉米、惠丰电源等一批企业建成投产，奎濉河治理等重点水利工程基本竣工；华电宿州生物质能发电、信诚化工、金黄庄煤矿建设进展顺利；汇源果汁、中粮集团环氧乙烷、雨润生猪屠宰、博洋纺织等项目开工建设；泗许高速公路宿州段用地已经国务院批准，宿淮铁路、钱营孜煤矿、森发纸业分别通过国家和省发改委核准；华电二期具备评优核准条件。这些重大项目的顺利推进，确保了有效投入的持续扩大，推动了经济结构的持续改善。

（五）城乡建设全面提速，人居环境日益改善。编制完成宿城总体规划局部调整、城市近期建设、汴河新区和人民路开发等规划。城市重点工程建设加快推进，市区基础设施建设投入完成5亿元，淮海南路、西昌南路等12条道路建成通车，拂晓大道北延、西昌路北延等4条道路正在施工，雪枫公园、银河公园对市民开放，博物馆、市立医院和皖北煤电总医院综合楼开工建设。城市综合开发势头强劲，市区旧城改造拆迁50万平方米，房地产开发竣工110万平方米，居住环境不断改善。围绕创建省级园林城市，投入1.5亿元，实施10项园林绿化工程，新增绿地面积200万平方米，绿地率提高5个百分点。城市净化、亮化、美化等综合整治扎实有效，垃圾处理场一期基本建成，市容市貌明显改观。四个县城基础设施建设步伐加快，县城规模逐步扩大，小城镇建设势头良好。

（六）社会事业长足进步，民主法制与政府自身建设得到加强。高度重视社会事业和民生问题，全市用于科技、教育、卫生、文化支出21.6亿元，社会保障支出12亿元，分别增长42%、32%。城镇新增就业岗位2.1万个，安置下岗失业人员再就业1万人。全市养老、失业、医疗、工伤等参保人员达到80万人。科技进步步伐加快，与合工大开展了产学研合作，萧县获“国家科技入户工程样板县”称号。“两基”成果持续巩固，教育质量不断提高，职业教育成效显著，教育资源整合取得积极进展。公共医疗卫生服务水平明显提升，第三次荣获“全国无偿献血先进城市”称号。文化体育事业加快发展，成功举办“江淮情”、“雪枫百年”文艺演出、书画晋京展和市第三届运动会等重大活动。计划生育、国土资源、环境保护等基本国策得到较好落实，性别比综合治理工作得到国家人口和计生委的认可与推广。“五五普法”深入开展，“平安宿州”建设扎实推进，大信访工作格局初步形成，应急管理体系逐步完善，安全生产形势总体平稳，产品质量和食品安全专项整治阶段性任务基本完成。支持驻宿部队建设，国防后备力量和人防建设进一步加强，实现“全国双拥模范城”五连冠。深入开展“两风”和机关效能建设，深化行政审批制度改革，政府办事程序进一步规范，办事效率和服务水平明显提高。审计监督力度加大，政府廉政建设不断加强。自觉接受人大法律监督和政协民主监督，人大代表议案、建议和政协委员提案全部办复。人事编制、民族宗教、统计、防震、气象、残疾人、老龄、新闻出版、外宣、外事侨务、对台、地方志、工青妇等各项事业都取得了新成绩。

各位代表，在推动经济发展的同时，我们把解决好关系群众切身利益的“生活难、看病难、上学难”等突出问题，作为推进社会和谐的重要切入点，共筹集资金7.2亿元，全面实施了十二项民生工程，400多万群众直接受益。农村居民最低生活保障制度基本建立，14万人纳入农村低保；3.3万名农村“五保户”按每人每年不低于1200元落实了供养标准；城镇未参保集体企业退休人员已全部领取基本生活费；农村新型合作医疗覆盖埇桥、砀山、泗县共计226万人，萧县、灵璧也已启动；城镇居民基本医疗参保人数达28.7万人；14万城乡特困群众得到医疗救助，对重大传染病人实行医疗救治和生活救助；城乡卫生服务体系建设、中小学危房改造、农村饮水安全工程取得积极进展，建设社区卫生服务中心、乡镇卫生院、村卫生室144个，消除中小学D类危房23.5万平方米，建成农村饮水安全工程134处；城乡义务教育经费得到有效保障，82.8万名学生享受“两免一补”；农村部分计划生育家庭奖扶资金及时发放。从十二项民生工程实施效果看，社会各界反响强烈，人民群众普遍欢迎，促进了社会和谐稳定。

各位代表，这些成绩的取得，是市委正确领导的结果，是市人大、市政协监督和支持的结果，是包括广大外来投资者在内的社会各界共同努力的结果，凝聚着全市人民的心血和智慧。在此，我谨代表市人民政府，向全市广大工人、农民、知识分子和离退休老同志，向各民主党派、工商联和人民团体，向驻宿解放军、武警官兵和政法干警致以崇高的敬意！向所有关心、支持、参与宿州建设和发展的同志们、朋友们，表示衷心的感谢！

在肯定成绩的同时，必须清醒地看到，我市经济社会发展中还面临着一些困难和问题。主要是：经济总量小，结构性矛盾尚未根本缓解，发展速度还不快；投资规模小，投资结构有待优化，综合实力不强；社会保障、就业、稳定等方面依然存在一些薄弱环节。对于以上问题，市政府将高度重视，坦诚面对，努力在发展中加以解决。

二、2008年发展目标

今年是全面贯彻党的十七大精神的起始之年，是实施“十一五”规划承上启下的关键一年。今年政府工作的总体要求是：全面贯彻党的十七大精神，高举中国特色社会主义伟大旗帜，以邓小平理论和“三个代表”重要思想为指导，深入贯彻落实科学发展观，按照“全面追赶、重点突破、局部超越”的总体要求，坚持以转变发展方式、加快结构调整为主线，以推进工业化、城市化为核心，深入实施五大战略，更加重视改善民生和促进社会和谐，加快实现“两个跃升、一个崛起”的奋斗目标。

今年的工作，我们将突出发展和民生两大主题，力争实现以下主要目标：地区生产总值增长12%；财政收入增长12%；固定资产投资和招商到位资金双超150亿元，分别增长30%、50%；全社会消费品零售总额增长16%；城镇居民人均可支配收入和农民人均纯收入均增长10%；城镇登记失业率控制在4.5%以内；人口出生率控制在13‰以内；单位生产总值能耗下降4%以上。

上述目标任务，是在全面分析当前形势、综合考虑多方面因素基础上安排的，与“十一五”规划、市第三次党代会提出的发展目标紧密衔接，充分考虑了可能和需要。

从可能看，我市已经具备了加快发展的条件。在外部环境上，国内整体经济高位平稳运行的态势没有改变，各类生产要素向中西部地区加快流动的趋势没有改变，国家促进中部地区崛起，省委、省政府支持皖北地区发展的战略没有改变，发展环境总体有利；在发展阶段上，进入“十一五”以来，全市生产总值始终保持两位数以上增长，预计在今年，第二产业在三次产业中的比重将首次超过一产，实现三次产业结构的历史性转折，人均生产总值将突破1000美元大关，迎来发展的重要拐点；在发展态势上，多年积累的能量正在加速释放，随着一批重大项目的核准和建设，发展的后劲更加充足；同时，各级干部应对复杂环境、驾驭工作全局、领导科学发展的能力正在不断提高。总体上，我市已经进入厚积薄发、加速跨越的新阶段，开始迈上持续较快增长的平台。

从需要看，虽然我市综合实力明显增强，但在省内人口总量靠前、经济总量居中、人均水平偏后的基本市情还没有根本改变，发展不够仍然是当前最大的矛盾。日益激烈的区域竞争，不允许我们放慢发展速度，求富思变的民心民意，不允许我们停步不前，我们必须坚持加快发展不动摇、不放松，既要好字优先，更要好中求快，在全市进一步掀起“大开放、大建设、大发展”的热潮，矢志不渝地打好“资源牌、文化牌、效能牌”，坚定不移地加快工业化、城镇化和农业产业化进程，放眼全局，拉高标杆，摒弃杂念，奋起直追，努力提升宿州在全省发展格局中的位次。

各位代表，发展才有实力，有实力才能为民造福。宿州当前正处在经济结构转型的关键时期，正处在爬坡过坎的紧要关口，快进则生，慢进则退，机不可失，时不我待。我们必须牢固树立强烈的发展意识、机遇意识和忧患意识，以敢于超越的勇气，不甘落后的志气，务求必胜的豪气，在不争议中发展，在不折腾中前进，在相对落后情况下加快赶超步伐。只有这样，才能不辜负全市600万人民的重托，宿州的未来才会大有希望！

三、推进又好又快发展，加速宿州崛起进程

不断增强经济实力，是全面追赶、奋力崛起的首要任务。必须抓住关键，破除瓶颈，保持经济快速发展的良好势头。

（一）强力组织工业突破，全面完成工业三年倍增计划。今年是全面完成工业三年倍增计划的决战决胜之年，必须集中精力、集中要素强攻工业，确保规模以上工业增加值增长26%以上，总量突破百亿大关，在全面完成工业三年倍增的基础上，启动工业总量“五年翻两番”计划。依托资源的广度、深度开发，依托现有企业的扩张带动，加快建设煤电能源、加工制造、农副产品深加工三个百亿元产业基地，突出发展煤炭、电力、纺织等八个主导行业，打造一批亿元项目，培植一批十亿元企业，形成一批销售收入超二十亿元的产业集群。加快企业内涵改造和外延扩张，重点抓好30个投资超3000万元的工业技改项目，力争全市实现产值超亿元企业50户，产值超5亿元企业10户，产值超10亿元企业2户。大力推动全民创业，加强中小企业、民营企业社会服务体系建设，催生和壮大一批成长性好、竞争力强的中小企业，力争规模以上企业超过400家。实施科技创新引导工程，增强科技创业中心孵化功能，建设30家省、市级企业技术中心，促进产业结构调整和技术升级。严格落实节能减排“问责制”，大力发展循环经济，加快推进节能工程、治污工程建设，重点抓好40户高耗能企业的节能减排工作。不断改善工业发展条件，有效缓解要素制约。努力完成142户国有、集体企业改制任务，积极培育上市资源。强化对工业发展的调度和考核，引导企业实施好科技创新、人才培养、企业管理、品牌培育四项工程。

（二）加速中心城市建设，强势推进城市管理上水平。以争创省级文明城市、园林城市和卫生城市“三城同创”为抓手，促进经济开发区、主城区和汴河新区“三区联动”发展，做大做强中心城市。坚持规划先行，完成主城区、新城区等区域规划和各类专项规划编制，确保城市发展健康有序。大力完善城市基础设施，抓好16条城市主干道路、4项市政公用工程和10处园林景观建设，提升城市功能。全面启动汴河新区建设，实施人民路区域综合开发，加快汴河北岸路网建设，加速新老城区对接；推进博物馆、体育馆、三角洲公园、沱河景观带和汴河大桥等标志性工程，打造新区发展亮点，营造现代化滨河生态城市风貌。加大主城区成片改造力度，完善西南片、道东片路网，提升城市出入口形象。继续推进城市洁净、绿化、美化、亮化工程，按照增绿量、建精品、创特色的要求，实现创建省级园林城市目标。加强城市精细化管理，落实城市管理领域相对集中处罚权工作，进一步完善两级政府、三级管理、四级网络的工作格局，构建市区联动、调控有力的城市管理长效机制。

（三）切实转变农业发展方式，增强农业和农村经济发展活力。坚持以发展现代农业为重点，促进农民持续增收，农村和谐稳定。把农产品加工业作为发展现代农业的主攻方向，围绕六大主导产业，强力推进农业产业化经营，大力扶持水果、粮食、畜牧、板材四大产业加工集群，力争粮食加工达到200万吨，水果加工达到100万吨，板材加工达到100万立方，提前实现“252”强龙工程目标，龙头企业营销收入超过百亿元，市级以上龙头企业达到130家，实现国家级龙头企业“零”的突破，产业化基地达到460万亩，农民合作经济组织达到500个。继续实施“双百工程”，大力发展“一村一品”为重点的特色高效农业。加强农产品质量安全检验监测体系建设和动物疫病防治，提高农产品质量安全水平。加大农业综合开发力度，加快农业机械化进程。加强新型农民培训，积极发展农村二、三产业和劳务经济。坚持以规划引导新农村建设，继续实施示范村镇饮水安全、户用沼气等工程。继续加强“村村通”道路建设，加快通乡公路改造，逐步消除乡村断头路。加大农村基础设施投入，强化农田水利基本建设，实施湖洼地治理、病险水库除险加固等重点水利工程，促进农村面貌有较大改观。深入推进农村综合改革，完善各项补贴政策，探索建立土地使用权市场化流动机制，稳步实施土地适度规模经营，加快集体林权、兽医管理、农技推广和种子管理体制改革步伐。

（四）扎实开展项目建设年活动，千方百计扩大投资。把项目建设作为推动又好又快发展的“引擎”，深入开展项目建设年活动，抓住新项目核准、在建项目推进、后续项目储备等关键环节，建立健全分工协作、调度管理、项目服务和考核奖惩机制，畅通重大项目建设绿色通道，全力以赴多上项目、上大项目、上好项目。积极创造条件，认真落实与皖能集团、皖北煤电集团、省出版集团的战略合作框架协议，加快推进一揽子项目的实施。突出抓好重大工业项目，力争汇源果汁、信诚化工、防爆电气、华电宿州生物质能发电等项目建成投产；加快钱营孜及金黄庄煤矿、中粮集团环氧乙烷、元邦精密带钢、博洋纺织、雨润生猪屠宰等项目建设进度；开工建设森发纸业、龙王庙和朱楼煤矿、天瑞及珍珠水泥、冠军陶瓷等项目；力争华电二期上报国家核准，积极做好汇源“大代小”、皖能宿州发电等项目前期工作。着力推进重大基础设施项目建设，加快泗许高速公路宿州段、303省道等世行项目建设，积极推进明徐高速公路宿州段、济祁高速公路宿州段前期工作，构建宿州新一轮公路网架。开工建设宿淮铁路、22万伏宿北、泗县、萧县输变电工程，全力支持京沪高速铁路宿州段建设，加速推进新汴河、浍河航道改造。积极争取国债资金，加快公共卫生体系、文化教育设施项目建设。加强项目储备，顺应国家政策导向，谋划一批投资5亿元、10亿元以上的战略性项目，确保“6＋3”项目库储备规模保持在千亿元以上，争取更多项目列入省“861”行动计划。

（五）全面深化东向发展，再掀对外开放新高潮。把招商引资作为扩大对外开放的突破口，深入推进东向发展，继续密切与长三角地区的经济联系，加大在苏浙沪的招商力度，加强与徐州经济圈的协作沟通，提高对外开放水平。严格落实招商引资工作目标责任制，建立以奖为主的激励机制，充分调动全市上下招商引资的积极性。进一步整合招商资源，做到招商力量向重大项目集中、园区资源向重大项目汇聚、考核奖惩向重大项目倾斜，更大规模地积累发展要素。围绕优势产业的延伸配套和新兴产业的培育壮大，强化产业招商和专业招商。积极参加国内重大招商活动，适时组织专项项目推介会，大力发展会展经济，认真办好第三届中国宿州灵璧石文化节，扩大对外宣传，提高宿州的知名度和影响力。加快转变外贸增长方式，重点建设3个省级出口基地，稳步扩大农产品、板材、纺织服装等特色产品出口规模，力争全年出口总额增长15%以上。完善市县两级行政服务中心功能，建立健全投诉受理快速联动机制，开展行政审批权相对集中试点工作，不断优化经济发展环境。

按照“强机制、壮产业、提形象、大发展”的基本思路，把开发区建设和产业发展结合起来，突出主导产业和优势项目，做好功能分区、产业集聚等专项规划，强化功能性配套设施建设，实现资源利用与外部资金、技术有效衔接，促进项目向开发区集中、园区向专业化发展、产业向上下游延伸，提升开发区的竞争力。结合城市规划调整，用足用好建设用地置换政策，拓展工业发展空间。争取更多的用地指标，有效解决现有开发区容量不足的问题。

（六）加快提升现代服务业，培育新的经济增长点。把发展服务业放在产业结构调整的突出位置，切实抓好机制创新、政策落实和载体培育，加快上规模、上水平，使服务业增加值占GDP的比重每年提高1～2个百分点。加快发展现代物流业，推进皖北煤炭物流、光彩城物流中心、开发区物流园、华夏物流配送中心等一批重点项目建设，打造现代物流平台。做大做强商贸流通业，合理规划商业布局，积极推进中心商圈建设，抓好一批大型商贸设施建设，积极引进大型百货商店、家电专业店和高档酒店，力争家乐福、大润发等知名零售企业落户宿州。重点培育一批区域性特色市场。继续实施“万村千乡”市场工程，逐步建立集生活消费、农资销售和农副产品收购为一体的农村市场网络。积极发展金融服务业，加强社会信用体系建设，优化金融生态环境。继续抓好徽商银行入驻，加快市区、灵璧、泗县信用社组建农村合作银行步伐，认真做好央行票据兑付工作，启动政策性农业保险试点，完善中小企业融资担保体系，构建政府、企业、金融机构和担保中心协调互动的平台。积极发展咨询评估、法律服务等现代服务业。进一步规范房地产市场，促进房地产业健康发展。深入挖掘汉文化内涵，大力开展旅游宣传促销和交流合作，精心打造皇藏峪等一批旅游品牌。

（七）坚持分类指导，做大做强县域经济。把振兴县域经济作为统筹城乡发展的基础性工程，鼓励和支持各县区发挥比较优势，按照以民营经济为主体、招商引资为重点、县城和中心镇为依托的总体发展思路，加速形成一二三产业协调发展、各具特色的现代县域经济。支持埇桥区提升经济首位度，争当东向发展排头兵。支持萧砀大力发展煤电产业、水果加工和旅游业，推进萧县向全国小麦加工第一大县、砀山向全国水果加工第一大县迈进。支持灵泗积极发展农副产品精深加工和劳动密集型产业，进一步蓄积发展能量，提升综合实力。加快四个县城建设，各县要以争创省级文明县城为目标，不断改善环境，提升形象，完善城镇规划体系，做大县城规模，集聚资金、人才、信息等各类要素，提高承载和经济辐射力。继续落实好扩大县域经济社会管理权限相关政策，加大对县域经济的财政、金融支持力度，增强县域经济的自我发展能力。支持县区对毗邻地区政策实行“拿来主义”，鼓励大胆创新，加快“跟跑”步伐，实现在省内的争先进位。制定完善乡镇发展扶持政策，在小城镇建设、项目安排、财政分配等方面向乡镇倾斜。抓住小城镇总体规划调整修编机遇，结合“空心村”整治，解决好乡镇工业园区的要素制约，着力打造一批工业增加值过亿元、财政收入过千万元的经济强镇，推动县域经济加快发展。

四、高度关注民生，着力构建和谐宿州

社会和谐稳定、百姓安居乐业，是广大人民的共同愿望，也是政府工作的重要内容。我们将始终坚持以人为本，认真解决好人民群众最关心、最直接、最现实的利益问题，切实让改革发展的成果更好地体现到提高人民生活水平和质量上。

（一）加快发展各项社会事业。强化政府对义务教育的责任，全面实施城乡免费义务教育，加快学校布局调整步伐，着力改善农村办学条件,促进义务教育均衡发展。规范发展民办教育，大力发展职业技术教育，支持宿州学院、宿州职业技术学院等高校建设。深化医疗卫生体制改革，全面推行新型农村合作医疗，完善农村三级医疗卫生体系，努力提高公共卫生服务能力和水平。突出抓好社区和乡镇综合文化站、文化信息资源共享、农村电影放映和送书下乡等工程，组织开展“百戏进百乡”活动。深入宣传“和睦和衷、尚贤尚信、大志大气、决战决胜”的宿州精神，使之成为加快发展的内在动力和精神支撑。广泛开展全民健身活动，提高竞技体育水平。加强计划生育基层基础工作，强化流动人口管理，努力降低出生人口性别比。进一步加强土地管理，切实提高土地综合利用效率和土地开发效益。加强和谐社区建设，启动农村社区建设试点。认真开展第三次全国文物普查、第二次经济普查、第二轮修志和“十一五”规划实施情况中期评估。加强国防教育和国防后备力量建设，做好人民防空、民兵预备役和拥军优属工作。全面贯彻党的民族宗教工作基本方针。依法维护妇女儿童合法权益，积极发展残疾人事业和老龄事业。

（二）着力提升民生工作水平。按照“立足当前、着眼长远、考虑总体、惠及多数”的原则，加大对民生工程的投入力度，提高民生政策保障标准，扩大政策覆盖面。在现有基础上，新增广播电视村村通、高校和中职学校学生资助、大中型水库移民后期扶持、农村公路村村通、解决城市低收入家庭住房困难及贫困白内障患者复明等六项民生工程。进一步强化各级政府的落实责任，完善项目资金保障监管，把为民的实事办好、好事办实。坚持积极的就业政策，加强就业观念教育，完善困难群众就业援助制度，重点促进“零就业”家庭就业。加快建立覆盖城乡的社会保障体系，完善城乡居民最低生活保障制度，扩大养老、医疗、失业、工伤、生育等保险范围，基本实现城镇居民医疗保险全覆盖。加快城乡社会救助体系建设，强化防灾减灾工作，做好扶贫救济工作，扶持社会力量兴办福利和慈善事业。

（三）营造稳定和谐的社会氛围。加强和改进新形势下的群众工作和信访工作，深入做好矛盾纠纷排查调处工作，严格落实信访责任制，及时解决群众反映的实际问题。强化安全生产管理和监督，严格落实安全生产责任制，严防重特大事故发生。整顿规范市场秩序，加强产品质量和食品药品安全监管，保障人民群众健康安全。切实做好副食品市场供应，认真落实“菜篮子”行政首长负责制，维护价格水平相对稳定。完善应急管理体制机制，提高保障公共安全和处置突发事件的能力。抓好“五五”普法宣传教育。完成村居换届选举。深入开展平安创建活动，健全社会治安防控体系，加强城乡社区警务工作，依法防范、严厉打击各种违法犯罪活动，为发展创造良好的社会环境。

各位代表，今年是完成本届政府五年目标任务的最后一年。百舸争流的态势，全市人民的重托，对政府工作提出了更高的要求，我们必须切实加强政府自身建设，以建设服务型政府为目标，以提高行政效能、增强政府的公信力和执行力为着力点，大力推进体制创新和管理创新，全面提高科学行政、民主行政、依法行政水平。今年政府工作：

要在转变职能上有新突破。把精力更多地倾注到社会发展和管理上来，把财力物力更多地集中到公共服务上来，把政策的着力点更多地放到解决民生问题上来，真正把政府该管的事一定管好，能做的事一定做好。

要在提高效能上有新成效。坚持不懈地改进“两风”，进一步弘扬雷厉风行的作风，强化工作执行力和落实力，着力解决不作为、慢作为和乱作为等突出问题，不断促进各级机关工作效率更高，服务质量更优，运行流程更畅。

要在推进依法行政上有新作为。严格按照法定权限和程序行使权力、履行职责，依法实施公共服务和社会管理。主动接受人大的法律监督、政协的民主监督和社会各界的监督，广泛听取各民主党派、工商联和无党派人士的意见和建议，推进政府决策的科学化、民主化。

要在加强党风廉政建设上有新举措。加强政务公开，建立健全政府信息公开考核制度、社会评议制度和责任追究制度，确保政务规范、政事清明。认真履行政府廉政承诺，加大对腐败行为的查处力度，切实纠正损害群众利益的不正之风，以清正廉洁的良好形象取信于民。

全市广大公务员务必保持奋发有为的精神状态，坚决革除与又好又快发展不相适应的思维定势和工作方式，以义无反顾的决心、百折不挠的毅力、心无旁骛的信念，善于攻坚克难，敢于抓难碰硬，着力推进宿州在新起点上实现更大跨越！

各位代表！建设富裕、文明、和谐的新宿州，是全市600万人民的共同事业，是永无止境的长期任务，是政府光荣而神圣的使命。让我们高举中国特色社会主义伟大旗帜，紧密团结在以胡锦涛同志为总书记的党中央周围，在中共宿州市委的坚强领导下，全面落实科学发展观，齐心协力，与时俱进，开拓创新，扎实工作，为夺取全面建设小康社会的新胜利而努力奋斗！
